# Draft Genome Sequence of the Picocyanobacterium *Synechococcus* sp. Strain GFB01, Isolated from a Freshwater Lagoon in the Brazilian Amazon

**DOI:** 10.1128/genomeA.00876-15

**Published:** 2015-08-13

**Authors:** Pedro Ivo Guimarães, Tiago Ferreira Leão, Aline Grasielle Costa de Melo, Rommel Thiago Jucá Ramos, Artur Silva, Marli Fatima Fiore, Maria Paula Cruz Schneider

**Affiliations:** a Department of Biological Systems Engineering, Virginia Tech, Blacksburg, Virginia, USA; b Center for Marine Biotechnology and Biomedicine, Scripps Institution of Oceanography, University of California San Diego, La Jolla, California, USA; c Federal University of Pará, Institute of Biological Sciences, Laboratory of DNA Polymorphism, Belém, Pará, Brazil; d Center for Nuclear Energy in Agriculture, University of São Paulo, Piracicaba, São Paulo, Brazil

## Abstract

We present the draft genome of the cyanobacterium strain *Synechococcus* sp. GFB01, the first genome sequencing of this genus isolated from South America. This draft genome consists of 125 contigs with a total size of 2,339,812 bp. Automatic annotation identified several genes involved with heavy metal resistance and natural transformation.

## GENOME ANNOUNCEMENT

The morphologically defined genus *Synechococcus* is composed of simple unicellular forms that are up to 2.0 µm in size, with high genetic diversity, and distributed in both freshwater and marine environments (1). The highly diverse genotypes reveal adaptation to different ecological niches (2). Furthermore, the molecular evidence for the polyphyly of this genus (3–8) indicates that the current classification requires revision. Some strains, such as *S. elongatus* PCC 7942, are model organisms that could be used as host strains in several synthetic biology methods due to their easy genetic manipulation, unicellular form, natural competence, and relatively fast growth rate (>1 division per day) (9–13). Whole-genome sequencing of a large number of *Synechococcus* strains from different environments will provide a broad understanding of the variability of strain genomes and better insights to the present classification of the genus. Currently, only 15 complete and 15 incomplete genomes of *Synechococcus* are available in the NCBI GenBank (as of January 2015), but none of them are from South America. In this work, we present the draft genome of *Synechococcus* sp. GFB01, isolated in 2011 from the surface of the freshwater lagoon Lagoa dos Índios (00°1′52.9248″N 51°6′9.2118″W), located in the municipality of Macapá, Amapá state, northern Brazil. The isolation procedure was carried out using BG-11 medium (14) and cycloheximide (70 mg/L) to inhibit eukaryotic cell growth. The amount of heterotrophic contaminant bacteria was reduced using a procedure adapted from Su et al. (15) consisting of washing 1 mL of the culture with SDS 10% (vol/vol) and transferring the treated inoculum to a fresh medium. After total DNA extraction, a genomic library was constructed using the Ion Shear Plus kit (Life Technologies, Carlsbad, CA, USA) and sequenced on the Ion Torrent PGM using the Ion Torrent PGM sequencing 200 kit version 2 and Ion 318 chip kit version 2 (Life Technologies).

A total of 2,930,850 reads, with an average length of 230 bp and 228-fold average coverage, was assembled using the MIRA software version 3.0 (16) and the assembled contigs were curated using the LASERGENE software. The curated contigs were extended using the SSPACE-LongRead software using the default parameters (17). The final draft of the *Synechococcus* sp. GFB01 genome consisted of 125 contigs with a total size of 2,339,812 bp and a G+C content of 67.77%. The automatic annotation using the RAST annotation server (18, 19) identified 2,727 predicted coding regions and 15 predicted rRNA genes. Several genes involved with resistance to heavy metals, such as arsenic, cobalt, nickel, and copper, and genes involved with antibiotic resistance were identified. Moreover, genes responsible for the assembly and function of the type IV pili were also found, suggesting that this strain is naturally transformable, making the *Synechococcus* sp. GFB01 a good candidate for a host strain for several synthetic biology techniques.

### Nucleotide sequence accession numbers.

This whole-genome shotgun project has been deposited at DDBJ/EMBL/GenBank under the accession number LFEK00000000. The version described in this paper is the first version, LFEK01000000**.**
